# Determinants of proactive health behaviors in individuals at high risks of stroke: a structural equation model analysis

**DOI:** 10.3389/fpubh.2026.1759806

**Published:** 2026-02-20

**Authors:** Mengdi Wang, Yijie Pan, Lixue Hou, Xiaowei Su, Bing Yu, Nannan Li, Luhan Zhang, Xin Li, Mengxia Chen, Lingjuan Zhang

**Affiliations:** 1 Department of Emergency and Critical Care Medicine, The 961st Hospital of Joint Logistics Support Force, Qiqihar, China; 2 Education and Scientific Research Department of Clinical Nursing, The First Affiliated Hospital of Naval Medical University, Shanghai, China; 3 Department of Nursing, The 961st Hospital of Joint Logistics Support Force, Qiqihar, China; 4 Department of Outpatient Follow-Up, Beidaihe Rest and Rehabilitation Center of Joint Logistics Support Force, Qinhuangdao, China; 5 Department of Nursing, The First Medical Center of Chinese PLA General Hospital, Beijing, China

**Keywords:** influencing factors, individuals at high risks of stroke, proactive motivation model, proactive health behaviors, structural equation modeling

## Abstract

**Aims:**

To explore the influencing factors of proactive health behaviors in individuals at high risks of stroke based on the theory of the proactive motivation model, and to clarify the interrelationship among these variables.

**Methods:**

A cross-sectional study using the convenience sampling method was conducted in one community in Shanghai, China. Between November 2024 and February 2025, a total of 309 individuals at high risks of stroke completed the general information questionnaire, the Self-Rated Abilities for Health Practices scale, the Chronic Illness Resources Survey, the Risk Perception Questionnaire for individuals at high risks of stroke, the 16-item European Health Literacy Survey Questionnaire, and the self-designed Proactive Health Behavior Scale for individuals at high risks of stroke. Descriptive statistics, univariate analysis, correlation analysis, and path analysis were used to identify the influencing factors and mechanisms of proactive health behaviors.

**Results:**

The structural model showed good model fit. There was a positive correlation between proactive health behaviors with chronic illness resources, health literacy, risk perception of stroke, and self-efficacy. Risk perception of stroke and self-efficacy both exerted a partial mediation effect between these variables and proactive health behaviors.

**Conclusions:**

Chronic illness resources, health literacy, risk perception of stroke, and self-efficacy are key factors associated with proactive health behaviors among individuals at high risks of stroke.

## Introduction

Stroke is a major global health concern, ranking as the second leading cause of death and the primary cause of long-term disability. The global prevalence of stroke in 2021 was 93.82 million people (1). China's number of stroke patients ranks first in the world, with 4.09 million incident cases of stroke, 26.34 million prevalent cases of stroke, and 2.59 million deaths from stroke (2). Previous studies predict that compared with those in 2019, the incidence, prevalence, deaths, and disability-adjusted life years (DALYs) due to stroke in China in 2050 will increase by 55.58%, 119.16%, 72.15%, and 20.04%, respectively (3). It is reported that approximately 50.0% of stroke survivors develop permanent disabilities and experience limitations in mobility, vision, speech, and swallowing function (4). The incidence rates of cognitive impairment, depression, anxiety, and fatigue after stroke are 64%, 48%, 29%, and 47%, respectively (5–8). The economic burden of stroke in China included direct costs of 247.8 billion RMB and indirect costs of 704.4 billion RMB, imposing a dual pressure on both society and families (9).

The escalating burden of stroke in China is predominantly driven by the expanding pool of high-risk individuals (10). Research indicates that the stroke incidence in individuals at high risks of stroke is 7–10 times higher than that of the normal individuals (11). Alarmingly, the prevalence of stroke-related risk factors in China is staggeringly high: approximately 250 million individuals with hypertension, 130 million with diabetes, 160 million with dyslipidemia, 350 million smokers, 4.87 million with atrial fibrillation, 23.9 million individuals who have experienced transient ischemic attacks, and 240 million who are overweight or obese (12). The confluence of these widespread risk factors has not only expanded the pool of stroke-susceptible individuals but also underscores the urgency of implementing intervention measures.

Stroke is preventable and controllable. A prospective cohort study conducted in Sweden observed a 54 % reduction in the risk of stroke among women who adhered to five healthy lifestyle factors, while men had a 72 % reduced risk of stroke (13). Despite this known efficacy, current primary prevention strategies often fail to actively engage individuals at high risks of stroke. For example, research indicates that among individuals at high risks of stroke, the prevalence rates for physical inactivity, smoking, alcohol consumption, high-salt diet, and high-fat diet are 46.0%, 18.6%, 12.2%, 21.2%, and 24.6%, respectively (14). In addition, up to 50% of patients with chronic conditions such as hypertension and diabetes exhibit poor medication adherence (15). A central barrier is the pervasive lack of personal motivation to initiate and sustain long-term behavioral changes (16). Therefore, merely providing information is insufficient. Bridging this gap necessitates a fundamental paradigm shift from passive education to systematically cultivating proactive health behaviors. “Proactive Health” was first proposed by experts across multiple fields in China in 2015 (17), emphasizing that individuals bear primary responsibility for their own health (18). Proactive health behaviors, as the practical pathway to achieving proactive health, are a crucial link in translating this concept into actual health benefits.

Identifying determinants of proactive health behaviors in individuals at high risks of stroke is crucial for developing targeted and effective preventive strategies. However, existing research has not specifically focused on individuals at high risks of stroke. Furthermore, prior investigations into factors influencing health behaviors have often examined variables in isolation or through simple linear associations, without grounding them within a comprehensive theoretical framework. This fragmented approach fails to capture the complex, interdependent pathways through which psychological, social, and cognitive factors interact to shape health behavior (16, 19).

**Figure 1:**
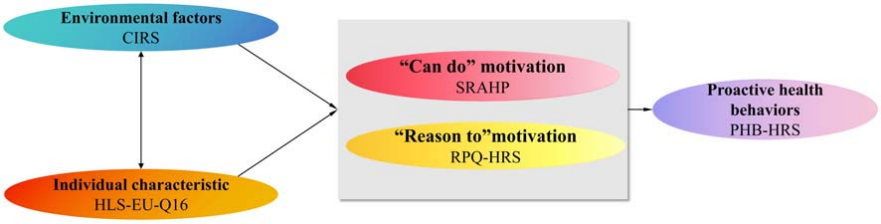
Hypothesized model.

To address these limitations, this study employs the proactive motivation model developed by Parker (20). The model posits that proactive behaviors are driven by three motivational states: “can do,” “reason to,” and “energized to,” which are in turn influenced by environmental and individual factors. We regard “can do” motivation as self-efficacy, “reason to” motivation as risk perception of stroke, environmental factors as chronic illness resources, and individual factors as health literacy. Given the multifaceted and theoretically mediated nature of these relationships, Structural Equation Modeling (SEM) offers a particularly suitable analytical framework. SEM enables the simultaneous examination of multiple direct and indirect effects within an integrated model, thereby providing a robust empirical foundation for designing theory-driven interventions. Therefore, in our study, SEM was employed to examine the relationships between these variables, with the theoretical framework presented in Figure 1.

Based on the above discussion, we propose the following hypotheses:

**Hypothesis 1:** self-efficacy, chronic illness resources, risk perception of stroke, and health literacy, significantly affect the proactive health behaviors of individuals at high risks of stroke.

**Hypothesis 2:** self-efficacy and risk perception of stroke play a mediating role between chronic illness resources, health literacy, and proactive health behaviors of individuals at high risks of stroke.

This study focuses on individuals at high risks of stroke and conducts an in-depth investigation of their proactive health behaviors based on the Proactive Motivation Model. It contributes to a deeper understanding of the relationships among self-efficacy, chronic illness resources, risk perception of stroke, and health literacy, as well as the potential pathways through which these variables may influence proactive health behaviors. Our study fills gaps in previous studies and provides a solid foundation for developing more effective guidance to enhance proactive health behaviors and reduce stroke incidence among individuals at high risks of stroke.

## Methods

### Study design and participants

This study was a cross-sectional study to explore the influencing factors and mechanisms of proactive health behaviors in individuals at high risks of stroke. From November 2024 to February 2025, using the convenience sampling method, 309 individuals at high risks of stroke were consecutively enrolled from a tertiary-level hospital in Shanghai, China. Inclusion criteria were as follows: (1) aged 18 years or older; (2) according to the Chinese Stroke Screening and Prevention Project (21), individuals at high risks of stroke are defined by the presence of three or more of the following risk factors: hypertension, dyslipidemia, diabetes, atrial fibrillation or valvular heart disease, a smoking history, obvious overweight or obesity, lack of exercise, and a family history of stroke; and (3) alert and able to communicate normally. The exclusion criteria were (1) mental illness or cognitive impairment, and (2) critical illnesses or other severe organic diseases.

### Sample size

According to Kendall's sample calculation method, the sample size should be at least 5–10 times the number of independent variables, and the sample size for structural equation modeling should not be less than 200 cases. This study involved a total of 40 independent variables. Considering a 10% sample loss rate, the required sample size was calculated to be between 210 and 420, and the actual sample size in this study was 309.

### Questionnaire design

#### Demographic and clinical information

Demographic information includes gender, age, marital status, education, residence status, occupation, monthly income, current smoking, and physical inactivity. Clinical information includes hypertension, dyslipidemia, diabetes, obesity or overweight, atrial fibrillation, and family history of stroke. The research team convened to deliberate on formulating a comprehensive survey questionnaire.

#### Self-rated abilities for health practices scale (SRAHP)

The Chinese version of the SRAHP was utilized in this study (22). The scale includes four dimensions, namely nutrition, psychological comfort, exercise, and health responsibility, comprising a total of 28 items. Employing a Likert 5-point rating scale, the total score ranges from 0 to 112, with higher scores indicating higher self-efficacy. In our study, the Cronbach's α of SRAHP was 0.964.

#### Chronic illness resources survey (CIRS)

The Chinese version was translated by Zhong Huiqin et al. (23). The CIRS consists of six dimensions: health care team, neighborhood or community, family and friends, personal, organizational structure, and media and policy, with a total of 22 items. The participants were instructed to evaluate their experiences over the past 6 months employing a 5-point Likert scale, ranging from 1 (never) to 5 (always). The total score of the scale ranges from 19 to 95 points, with higher scores indicating greater chronic disease resource utilization. In our study, the Cronbach's α of CIRS was 0.950.

#### Risk perception questionnaire for individuals at high risks of stroke (RPQ-HRS)

The questionnaire was developed by Ren Hui and comprises five dimensions: perceived susceptibility, perceived symptoms, perceived risk factors, perceived risk control, and perceived severity, totaling 30 items. Each item employs a 5-point Likert scale, scored from 1 (strongly disagree) to 5 (strongly agree). The total score ranges from 30 to 150 points, assessing the perceived risk of stroke among high-risk individuals. Higher scores indicate greater perceived risk of stroke (24). In our study, the Cronbach's α of RPQ-HRS was 0.972.

#### 16-item European health literacy survey questionnaire (HLS-EU-Q16)

This questionnaire comprises three dimensions: health care, disease prevention, and health promotion, with a total of 16 items. Each item was categorized as “very easy,” “easy,” “difficult,” or “very difficult.” “Very easy” and “easy” were scored as 1 point, “very difficult” and ‘difficult' were scored as 0 points, and “don't know/refused” was coded as missing. Health literacy levels are categorized as inadequate (0–8), problematic (9–12), and adequate (13–16) based on total scores (25). In this study, the Cronbach's α of HLS-EU-Q16 was 0.930.

#### Proactive health behavior scale for individuals at high risks of stroke (PHB-HRS)

This scale was developed by our research team and has passed reliability and validity tests. It includes six dimensions: symptom awareness, health responsibility, resource utilization, daily habits, overcoming obstacles, and information identification, with a total of 31 items (26). Each item uses a 5-point Likert scale, with scores ranging from 1 (never) to 5 (always). The total score range is 31 to 155 points. In this study, the Cronbach's α of PHB-HRS was 0.962.

### Data collection

The questionnaire survey was conducted with the consent of the relevant departments and sections of the hospital. Three trained researchers conducted participant screening by reviewing medical records or by interviewing nurses in inpatient wards and outpatient clinics. The questionnaire was created using “Wenjuanxing”, an electronic questionnaire platform. Prior to the distribution of the questionnaire, the purpose of the study was explained in detail to the participants, and their consent was obtained. The inquiry was conducted with strict confidentiality and anonymity, ensuring the privacy and anonymity of the respondents. The investigators provided guidance to the participants in the completion of the questionnaire. Participants who were unable to complete the questionnaire independently, the investigators completed it based on the participants' statements. The questionnaire was designed to require between 10 and 20 min to complete. After the questionnaires were collected, a database was established using EpiData 3.1 software, and double verification was performed to ensure the accuracy and completeness of the data.

### Ethical considerations

This study was approved by the Ethics Committee of Changhai Hospital (ethics approval number: CHEC2025-058). All participants signed written informed consent forms. This study complies with the Declaration of Helsinki and relevant Chinese regulations. All responses are anonymous and are used solely for academic research purposes. Participants were informed that they have the right to withdraw from the study at any time without penalty.

### Statistical analysis

Data analysis was performed by SPSS software version 26 using descriptive statistics (frequency, mean, standard deviation, and percentage). Group comparisons for normally distributed variables were conducted using independent-sample *t*-tests or one-way ANOVA; for non-normally distributed data, the Mann–Whitney *U* or Kruskal–Wallis tests were used. The significance level of *p* < 0.05 was considered. Pearson correlation coefficients were calculated to assess relationships among self-rated abilities for health practices, chronic illness resources, health behavior motivation, health literacy, and proactive health behaviors. For designing and fitting the model, the structural equation modeling method was used. The SEM model was evaluated using the following indices: Chi-Square Degrees of Freedom Ratio (*X*^2^/*df* ) <3.0, Goodness-of-Fit Index (GFI) >0.90, Tucker-Lewis Index (TLI) >0.90, comparative fit index (CFI) >0.90, incremental fit index (IFI) >0.90, normed fit indices (NFI) >0.90, and root mean square error of approximation (RMSEA) <0.08, indicating that the model has a high degree of fit.

## Results

### Participants characteristics and univariate analysis of differences in PHB-HRS

A total of 309 questionnaires were distributed and returned, 301 valid questionnaires remained, with an effective response rate of 97.4%. The average age of the participants was 38.04 ± 20.50 years, comprising 118 males (39.2%) and 183 females (60.8%). More than half (58.1%) held a Bachelor's degree or higher. The results of univariate analysis showed that there were significant differences in PHB-HRS scores among individuals at high risks of stroke with different age, education, marriage status, monthly income, and those with or without hypertension, dyslipidemia, diabetes, smoking, and family history of stroke (*P* < 0.05). Additional characteristics are presented in Table 1.

**Table 1:** Sample characteristics and univariate analysis of differences in PHB-HRS (*N* = 301).

**Variable**	**Frequency (*n*)**	**Constituent ratio (%)**	**PHB-HRS (M ± SD)**	***t/F***	***P***
**Gender**					
Male	118	39.2	107.33 ± 21.92	0.561	0.575
Female	183	60.8	105.84 ± 22.96		
**Age (years)**					
18–40	175	58.1	109.55 ± 23.40	4.201	**0.016**
41–60	66	21.9	102.88 ± 22.13		
>61	60	20.0	101.20 ± 18.87		
**Education**					
Illiterate/elementary school	42	14.0	71.21 ± 8.92	151.317	**<0.001**
Middle school	49	16.3	93.10 ± 13.16		
High school	35	11.6	101.40 ± 12.19		
Bachelor or above	175	58.1	119.61 ± 15.83		
**Marriage status**					
single	125	41.5	102.52 ± 20.86	4.817	**0.009**
Married	162	53.8	110.06 ± 23.50		
Divorced/Widowed	14	4.7	99.21 ± 18.83		
**Residence**					
Urban	215	71.4	106.49 ± 23.23	0.085	0.933
Rural	86	28.6	106.26 ± 20.82		
**Residency status**					
Living alone	35	11.6	103.20 ± 23.64	−0.900	0.369
Not living alone	266	88.4	106.85 ± 22.39		
**Occupation**					
Manual labor	138	45.8	106.55 ± 23.56	0.300	0.825
Brain labor	60	19.9	106.82 ± 20.79		
Unemployed	63	20.9	107.65 ± 23.28		
Retired	40	13.3	103.45 ± 20.70		
**Monthly income (RMB)**					
<1000	29	9.6	83.21 ± 20.74	22.785	**<0.001**
1000–3000	74	24.6	99.84 ± 21.48		
3000–5000	98	32.6	108.45 ± 17.38		
>5000	100	33.2	116.04 ± 22.19		
**Obese or overweight (BMI**≥**28kg/m**^2^**)**					
No	262	87.0	106.64 ± 22.37	0.437	0.662
Yes	39	13.0	104.95 ± 23.85		
**Hypertension**					
No	204	67.8	116.92 ± 17.31	15.904	**<0.001**
Yes	97	32.2	84.34 ± 15.03		
**Dyslipidemia**					
No	213	70.8	114.89 ± 18.06	12.500	**<0.001**
Yes	88	29.2	85.92 ± 18.85		
No	252	83.7	111.17 ± 20.08	9.430	**<0.001**
Yes	49	16.3	82.00 ± 18.38		
**Atrial fibrillation**					
No	281	93.4	106.85 ± 22.66	1.249	0.213
Yes	20	6.6	100.35 ± 20.10		
**Current smoking**					
No	232	77.1	112.29 ± 20.30	9.426	***P*** **<** **0.001**
Yes	69	22.9	86.68 ± 18.07		
**Physical inactivity**					
No	175	58.1	107.76 ± 22.74	1.215	0.225
Yes	126	41.9	104.56 ± 22.19		
**Family history of stroke**					
No	273	90.7	107.26 ± 22.34	2.035	**0.043**
Yes	28	9.3	98.21 ± 23.12		

M, mean; SD, standard deviation; *t*, values of *t*-test; *F*, values of ANOVA; PHB-HRS, Proactive Health Behavior Scale for individuals at high risks of stroke. Values in bold indicate statistical significance (*P* < 0.05).

### Scores and correlation of variables

The total scores for PHB-HRS, CIRS, SRAHP, RPQ-HRS, and HLS-EU-Q16 were 106.42 ± 22.53, 64.73 ± 14.66, 73.09 ± 19.88, 84.34 ± 23.23, and 10.96 ± 5.20, respectively. Pearson's correlation analysis revealed that PHB-HRS was significantly positively correlated with CIRS (*r* = 0.688; *P* < 0.01), SRAHP (*r* = 0.672; *P* < 0.01), RPQ-HRS (*r* = 0.633; *P* < 0.01), and HLS-EU-Q16 (*r* = 0.625; *P* < 0.01). The specific correlations are shown in Table 2.

**Table 2:** Mean ± SD and spearman correlation analysis of each variable (*N* = 301).

**Variable**	**M ± SD**	**PHB-HRS**	**CIRS**	**SRAHP**	**RPQ-HRS**	**HLS-EU-Q16**
PHB-HRS	106.42 ± 22.53	1.000	–	–	–	–
CIRS	64.73 ± 14.66	0.688^**^	1.000	–	–	–
SRAHP	73.09 ± 19.88	0.672^**^	0.530^**^	1.000	–	–
RPQ-HRS	84.34 ± 23.23	0.633^**^	0.541^**^	0.543^**^	1.000	–
HLS-EU-Q16	10.96 ± 5.20	0.625^**^	0.517^**^	0.521^**^	0.496^**^	1.000

M, mean; SD, standard deviation; RPQ-HRS, Risk perception questionnaire for individuals at high risks of stroke; CIRS, chronic illness resources survey; SRAHP, Self-rated abilities for health practices scale; PHB-HRS, Proactive health behavior scale for individuals at high risks of stroke; HLS-EU-Q16, 16-item European health literacy survey questionnaire.

^**^P < 0.001.

### Structural equation model of factors

**Figure 2:**
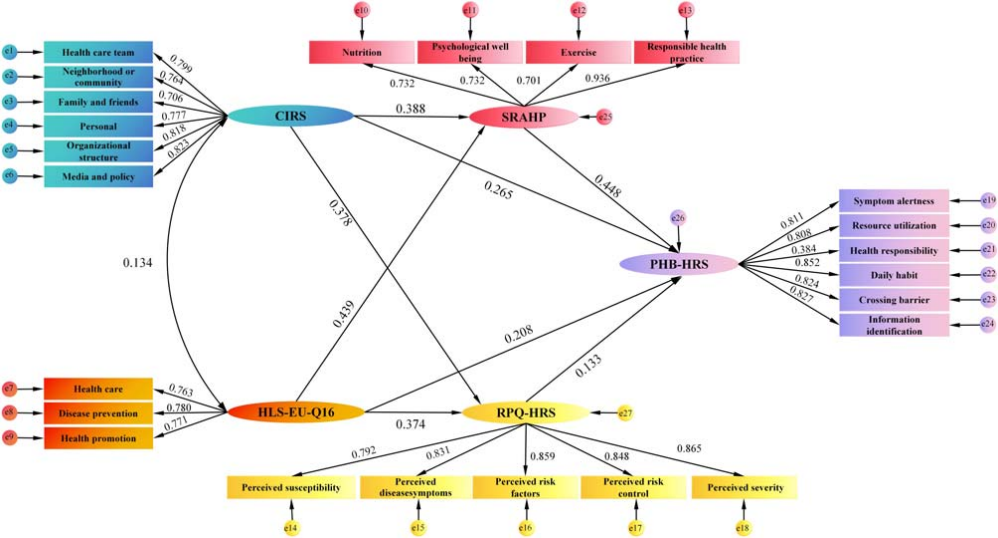
Test of the hypothesized model.

A structural equation model was constructed using AMOS based on the preliminary hypotheses. The model fit results indicated that the chi-square degree of freedom ratio was 1.394, GFI was 0.913, TLI was 0.977, CFI was 0.980, IFI was 0.980, NFI was 0.934, and RMSEA was 0.036, indicating that the model fits well. As shown in Figure 2, all standardized path coefficients were meaningful (*P* < 0.05). From Table 3, CIRS was a positive predictor of SRAHP, RPQ-HRS, and PHB-HRS (β = 0.388, 0.378, and 0.265, respectively; all *P* < 0.05). HLS-EU-Q16 also positively predicted RPQ-HRS, SRAHP, and PHB-HRS (β = 0.374, 0.439, and 0.208, respectively; all *P* < 0.05). Furthermore, SRAHP and RPQ-HRS positively predicted PHB-HRS (β = 0.448 and 0.133, respectively; both *P* < 0.05). Specifically, SRAHP acts as a mediator between CIRS and PHB-HRS, as well as between HLS-EU-Q16 and PHB-HRS. RPQ-HRS mediates between CIRS and PHB-HRS, as well as between HLS-EU-Q16 and PHB-HBS.

**Table 3:** Path hypothesis test results (*N* = 301).

**Path**	**Estimate**	***SE***	***P***	**95% *CI***
CIRS → SRAHP	0.388	0.080	0.010	0.212–0.533
SRAHP → PHB-HRS	0.448	0.064	0.011	0.309–0.567
CIRS → PHB-HRS	0.265	0.053	0.012	0.158–0.383
CIRS → RPQ-HRS	0.378	0.075	0.009	0.211–0.513
HLS-EU-Q16 → SRAHP	0.439	0.081	0.009	0.288–0.603
HLS-EU-Q16 → RPQ-HRS	0.374	0.074	0.006	0.245–0.552
RPQ-HRS → PHB-HRS	0.133	0.052	0.010	0.038–0.251
HLS-EU-Q16 → PHB-HRS	0.208	0.067	0.013	0.069–0.342
CIRS → SRAHP → PHB-HRS	0.174	0.043	0.009	0.072–0.260
HLS-EU-Q16 → SRAHP → PHB-HRS	0.197	0.047	0.004	0.127–0.344
HLS-EU-Q16 → RPQ-HRS → PHB-HRS	0.050	0.022	0.005	0.017–0.116
CIRS → RPQ-HRS → PHB-HRS	0.050	0.021	0.007	0.015–0.101

RPQ-HRS, Risk Perception questionnaire for individuals at high risks of stroke; CIRS, Chronic illness resources survey; SRAHP, Self-Rated abilities for health practices scale; PHB-HRS, Proactive health behavior scale for individuals at high risks of stroke; HLS-EU-Q16, 16-item European health literacy survey questionnaire.

## Discussion

In the era of comprehensive health, citizens are recognized as the primary stewards of their own well-being (27, 28). Guiding individuals at high risks of stroke to actively adopt healthy behaviors is particularly important for preventing stroke. The proactive motivation model was employed in this study to investigate the influencing factors and underlying mechanisms of proactive health behaviors among individuals at high risks of stroke. In this study, the total proactive health behaviors score among individuals at high risks of stroke was 106.42 ± 22.53 points, with a score rate of 68.66%, indicating substantial room for improvement. Furthermore, statistically significant positive correlations were identified between self-rated abilities for health practices, chronic illness resources, risk perception of stroke, health literacy, and proactive health behaviors. Additionally, self-efficacy and risk perception of stroke mediated the relationship between chronic illness resources, health literacy, and proactive health behaviors. These insights provide a practical pathway for nursing managers to apply theoretical insights in tangible efforts to enhance proactive health behaviors among individuals at high risks of stroke.

In this study, the results of the univariate analysis indicated some differences in demographic factors in terms of the level of proactive health behaviors among individuals at high risks of stroke. Younger adults (aged 18–40 years) demonstrated higher proactive health behaviors than older groups (aged 41–60 and ≥61 years), and higher education was positively correlated with greater engagement in proactive health behaviors, likely due to better cognitive ability, health information access, and comprehension of health knowledge (29). Married individuals also exhibited higher proactive health behaviors, possibly owing to spousal support and mutual health monitoring (30, 31). Furthermore, higher household income was associated with higher Proactive health behaviors, consistent with greater material resources enabling better nutrition, exercise, and regular health screenings. These findings align with Erik et al.'s (32) Mendelian randomization study, which demonstrated that a 10% increase in income was associated with decreased rates of depression, mortality, smoking history, and BMI. Individuals at high risks of stroke with hypertension, hyperlipidemia, diabetes, and a family history of stroke generally exhibit lower levels of proactive health behaviors. This aligns with findings from Jin et al. (33), indicating that individuals with chronic conditions (e.g., hypertension, diabetes) display lower health behavior levels, highlighting a critical gap between clinical risk and preventive behavior.

We found that proactive health behaviors among individuals at high risks of stroke showed significant positive correlations with their chronic illness resources, risk perception of stroke, self-efficacy, and health literacy, which supports Hypothesis 1. Specifically, greater chronic illness resources are associated with higher levels of proactive health behaviors. Adequate chronic disease resources provide objective guarantees and external impetus for individuals to practice proactive healthy behaviors (34, 35). This finding is consistent with previous studies indicating that illness resources were associated with self-care behavior among patients with chronic illnesses such as diabetes, hypertension, heart disease, and hyperlipidemia (36, 37). Risk perception of stroke positively influences proactive health behaviors. According to the protection motivation theory (PMT), this may stem from perceived health threat reflecting individuals' awareness and concern about potential health risks or harm, where moderate threat perception can motivate proactive health behaviors. This result aligns with earlier research demonstrating that heightened risk awareness drives preventive engagement (38, 39). Individuals with high self-efficacy are more inclined to engage in proactive health behaviors. A systematic review explicitly states that integrating self-efficacy into behavior change intervention helps sustain the long-term maintenance of health behaviors (36). Health literacy is positively correlated with proactive health behaviors. Prior studies indicate that individuals with higher health literacy are better equipped to identify stroke risk factors, understand the value of healthy behaviors, and actively adopt measures such as balanced nutrition, regular exercise, and routine health monitoring (40, 41).

The mediation analysis underscores the significant role of self-efficacy and risk perception of stroke in linking chronic illness resources and health literacy to proactive health behaviors, thereby supporting Hypothesis 2. Our findings demonstrate that both chronic illness resources and health literacy exert robust positive effects on self-efficacy and stroke risk perception, which in turn facilitate the adoption of proactive health behaviors. Individuals with greater health literacy and better access to health support show stronger confidence in engaging in proactive health behaviors, along with a more accurate understanding of stroke risks. This awareness motivates them to adopt proactive health behaviors for improved well-being (42–44). Our findings align with and extend prior research. Recent studies have similarly identified that self-efficacy is a critical mediator between health literacy and health behaviors across various chronic conditions (45). Furthermore, research by Gye et al. demonstrated that risk perception mediates the relationship between health literacy and disease prevention behaviors (46). Notably, chronic illness resources and health literacy are not independent but interact dynamically to shape self-efficacy and risk perception of stroke (47). Higher health literacy equips individuals to more effectively seek, interpret, and utilize available chronic disease resources, thereby amplifying the practical utility of these resources. Conversely, abundant chronic disease resources (e.g., health education, medical services, and regular follow-ups) can enhance health literacy by providing repeated and contextualized learning opportunities.

### Implications for nursing practice and recommendations for future research

Our findings provide practical support for the proactive motivation model and validate the proposed research hypotheses. Nursing practitioners should design individualized proactive health behavior intervention strategies tailored to the demographic characteristics (such as age, education level, and marital status) of individuals at high risks. While enhancing the health literacy and chronic disease resources among individuals at high risks of stroke, it is essential to strengthen their awareness of stroke risk and sense of self-efficacy. These targeted interventions can effectively promote proactive health behaviors, thereby improving stroke prevention and management effectiveness.

Future research should extend these findings through multicenter longitudinal studies to clarify the temporal and causal pathways linking chronic disease resources, health literacy, self-efficacy, stroke risk awareness, and proactive health behaviors. Furthermore, subgroup analyses focusing on specific individuals at high risks of stroke (e.g., different educational status, multiple chronic conditions, and a family history of stroke) are needed to identify population-specific determinants and refine priorities for personalized interventions.

### Limitations

This study has several limitations. First, the cross-sectional design makes it difficult to establish causal relationships between variables and fails to capture the dynamic evolution of proactive health behaviors among individuals at high risks of stroke. Second, all participants were recruited from a single healthcare institution, which limits the representativeness of the sample and calls for caution when generalizing the findings. Third, the voluntary recruitment approach may have introduced selection bias, as individuals more willing to participate might possess greater health knowledge or more proactive health behaviors.

## Conclusions

This study demonstrates that chronic illness resources, health literacy, self-efficacy, and risk perception of stroke are significantly positively correlated with proactive health behaviors among individuals at high risks of stroke. Furthermore, both chronic illness resources and proactive health behaviors among individuals at high risks of stroke, as well as the relationship between health literacy and proactive health behaviors among individuals at high risks of stroke, are mediated by self-efficacy and risk perception of stroke. This mechanism demonstrates that stroke prevention and control measures should not only provide resources and health education but also focus on cultivating individuals' self-management capabilities and promoting their rational understanding of disease risks, thereby promoting proactive health behaviors in a multidimensional and comprehensive manner to ultimately reduce the risk of stroke incidence effectively.

## Data Availability

The raw data supporting the conclusions of this article will be made available by the authors, without undue reservation.
